# The Effect of Learning on Feedback-Related Potentials in Adolescents with Dyslexia: An EEG-ERP Study

**DOI:** 10.1371/journal.pone.0100486

**Published:** 2014-06-20

**Authors:** Dror Kraus, Tzipi Horowitz-Kraus

**Affiliations:** 1 Department of Pediatric Neurology, Cincinnati Children's Hospital Medical Center, Cincinnati, Ohio, United States of America; 2 Pediatric Neuroimaging Research Consortium, Cincinnati Children's Hospital Medical Center, Cincinnati, Ohio, United States of America; 3 Edmond J. Safra Brain Research Center for the Study of Learning Disabilities, Faculty of Education, University of Haifa, Haifa, Israel; The Ohio State University, Center for Cognitive and Brain Sciences, Center for Cognitive and Behavioral Brain Imaging, United States of America

## Abstract

**Introduction:**

Individuals with dyslexia exhibit associated learning deficits and impaired executive functions. The Wisconsin Card Sorting Test (WCST) is a learning-based task that relies heavily on executive functioning, in particular, attention shift and working memory. Performance during early and late phases of a series within the task represents learning and implementation of a newly learned rule. Here, we aimed to examine two event-related potentials associated with learning, feedback-related negativity (FRN)-P300 complex, in individuals with dyslexia performing the WCST.

**Methods:**

Adolescents with dyslexia and age-matched typical readers performed the Madrid card sorting test (MCST), a computerized version of the WCST. Task performance, reading measures, and cognitive measures were collected. FRN and the P300 complex were acquired using the event-related potentials methodology and were compared in early vs late errors within a series.

**Results:**

While performing the MCST, both groups showed a significant reduction in average reaction times and a trend toward decreased error rates. Typical readers performed consistently better than individuals with dyslexia. FRN amplitudes in early phases were significantly smaller in dyslexic readers, but were essentially equivalent to typical readers in the late phase. P300 amplitudes were initially smaller among readers with dyslexia and tended to decrease further in late phases. Differences in FRN amplitudes for early vs late phases were positively correlated with those of P300 amplitudes in the entire sample.

**Conclusion:**

Individuals with dyslexia demonstrate a behavioral and electrophysiological change within single series of the MCST. However, learning patterns seem to differ between individuals with dyslexia and typical readers. We attribute these differences to the lower baseline performance of individuals with dyslexia. We suggest that these changes represent a fast compensatory mechanism, demonstrating the importance of learning strategies on reading among individuals with dyslexia.

## Introduction

Dyslexia is a neurodevelopmental disorder characterized by difficulties in accurate and/or fluent word recognition and by poor spelling and decoding abilities. It is the most common specific learning impairment with an estimated prevalence of 5–6% among school-aged children in the United States. It is believed that dyslexia is mainly caused by a deficit in the phonological component of language [1].

Despite being a specific learning impairment, dyslexia has been associated with a range of deficits that extend beyond the verbal domain. These include nonverbal deficits such as sequential, visuomotor, implicit and procedural learning [2], [3], [4], as well as in executive functions such as speed of processing and performance monitoring [5], [6], [7].

The Wisconsin Card Sorting Test (WCST) is a well-validated neuropsychological task that assesses both learning and executive functioning. It was developed by Grant and Berg in 1948 [8] and was originally introduced as a measure of frontal lobe function by Milner [9]. It has been validated in children in several age groups [10], [11] and has been used clinically in children with ADHD [10], [12], Pervasive Developmental Disorder [12], temporal lobe epilepsy [13] and traumatic brain injury [14]. In the context of the WCST, learning consists of how fast a participant finds the card sorting rule and whether he is able to maintain it (see Methods below) [15]. Thereby, the main executive functions tested by the WCST are set-shifting (the ability to switch rules following a negative feedback) and working memory (the capacity to memorize previously tested rules and maintain a given rule). Other executive functions tested include attention, inhibition, and speed of processing [16].

Individuals with dyslexia do not seem to exhibit a consistent pattern of reading errors, which interferes with their potential to learn from previously made mistakes [17], [18]. In the context of the WCST, both adults and children with dyslexia were reported to commit more errors, complete fewer categories, and have longer reaction times as compared to age-matched typical readers [16], [19], [20].

Event-related potentials (ERPs) are stereotyped electrophysiological responses to a given stimulus. ERPs are measured using electroencephalography (EEG) and provide a non-invasive, objective and easily reproducible measure of processing by linking a series of stimuli and responses. Another advantage over behavioral measures is their sensitivity, i.e. the ability to measure processing of stimuli as well as providing information regarding cognitive processes/underlying mechanism even in the absence of a behavioral change [21].

Our previous study examined ERPs during performance of the Madrid Card Sorting Test (MCST), a computerzied version of the traditional WCST, in 12-year-old adolescents with dyslexia compared to age-matched typical readers [16]. Adolescents with dyslexia demonstrated smaller target-locked potentials. This was manifested by ERPs reflecting decreased attention/perception (smaller target-locked N100) and speed of processing and working memory abilities (demonstrated by smaller target-locked P300). This difference was particularly pronounced toward the end of a series [16].

While the previous study examined responses to stimulus processing (i.e., the presentation of cards in the MCST), the current study focused on cognitive processing following feedback perception (i.e. following the response). This can be assessed electrographically by measuring the feedback-related negativity event-related potential. Feedback-related negativity is a fronto-central negative potential, which is evoked 200–300 ms after an external feedback of an erroneous response is given. It originates presumably from the Anterior Cingulate Cortex [22]. Several studies linked the FRN to the P300 component, and referred to it as the FRN-P300 complex, which reflects learning ability [23], [24], [25]. The centro-parietal P300 cue-locked component provides complementary information about the learning process. It is thought to represent attentive processes [23] and the rapid shift of attention toward unexpected stimuli [26], [27], [28].

The effect of learning on cue (performance)-locked components has been the subject of debate. While some studies showed a decrease in both FRN and P300 amplitudes following learning [24], others found only P300 amplitudes to be decreased [25]. Another study [23] demonstrated changes in FRN-P300 amplitudes occurring in the course of a task. Comparing subjects who learned a task to those performing it for the first time, FRN amplitudes in the late phase were smaller than during early phases of the task in both groups, whereas P300 amplitudes in the late phase decreased only in trained subjects.

Such differences between early and late phases in a learning task were suggested to exist also in the MCST. Barcelo & Knight (2002) found that healthy adults were found to commit fewer errors in late than in early stages of MCST series (7–8 items per series) [29]. Subjects with frontal lobe dysfunction did not demonstrate a similar decrease in errors. The researchers also suggested that higher error rates during early responses in older vs. younger participants were due to working memory overload [29].

In the current study, we sought to characterize learning within MCST series among adolescents with dyslexia by examining FRN and P300. Specifically, we sought to monitor the evolution of these markers in early vs late trials. Assuming that readers with dyslexia have an impaired ability to learn from previous mistakes [18], we hypothesized that 1) Reaction times (RT) and error rates will decrease in late phases for both groups, but to a lesser extent in individuals with dyslexia; 2) In the early phases of individual series, individuals with dyslexia will exhibit decreased FRNs and P300 components compared to typical readers; 3) When comparing early and late phases of series, differences in FRN amplitudes will be correlated with differences in P300 amplitudes. These changes will be more pronounced among individuals with dyslexia; 4) FRN-P300 complex amplitudes will be associated with speed of processing, working memory and reading scores.

## Methods

### 2.1. Participants

All participants gave their informed written assent and their parents provided informed written consents prior to inclusion in the study. The experiment was approved by the University of Haifa Ethical Committee (number 009\09\09) based on the principles of the Helsinki declaration. Fifty-eight adolescents participated in the current study: 27 individuals with dyslexia (16 males, mean age 12.84±0.55 years old) and 31 age-matched typical readers (18 males, mean age 12.8±0.47 years old). All participants were eighth-grade students from a junior high school in the center of Israel, who volunteered for the study.

Participants with dyslexia and typical readers did not differ in their nonverbal IQs [all were within the normal range (90 and above) *t*(57) = −1.934, n.s)] as measured by the TONI-III general ability task [30] and normal attention skills as measured by two visual screening tests. In these tests the participants had to scan a page and circle specific targets (e.g., the three digits “592” or a shape of a diamond) (after [31]). Attention abilities did not differ between the two [592:t (57) = −.553, n.s; diamond: t(57) = −.447 n.s]. All were native Hebrew speakers from a middle-class background, right-handed, displayed normal or corrected-to-normal vision in both eyes, and were found on screening to have normal hearing. None of the participants had a history of neurological or emotional disorders. Participants with dyslexia had been diagnosed at least two years prior to the study and were found to meet the criteria of dyslexia as described below. The experiments were carried out at the school. All participants were compensated with a special pen.

### 2.2 Behavioral and Experimental Measures

Prior to the MCST, several cognitive and reading measures were assessed in all participants (see Table 1). Participants were assigned to the dyslexia group if they achieved a standard score of <−1 on standard normative one-minute words and nonwords (or pseudowords) reading tests in Hebrew (reading speed and accuracy). Additional descriptive measures included fluency in an oral reading passage [32] and reading comprehension measures. The behavioral testing lasted 1.5 hours.

**Table 1:** Behavioral measures administered to all participants.

	Description
**Reading measures**	A. Decoding — One-minute words/nonwords test for children [32]
	B. Oral reading task for children [32].
	D. Comprehension [33].
**Cognitive measures**	A. Short term memory and capacity — Digit span subtest from the WAIS-III [34].
	B. Coding WAIS-III [34].
	C. Symbol search WAIS-III [34].
	D. Naming letters, objects and Rapid Alternate Stimuli naming (RAS) [modeled after [35], [36].
	E. Trial making test [37].

#### 2.2.1 The Madrid Card Sorting Test

Executive abilities were examined using the Madrid Card Sorting Test (MCST), a computerized version of the WCST adapted from Barcelo [38]. The MCST is a standardized tool that measures ERP components elicited during task-shifting. Stimuli were presented by Presentation® software (Neurobehavioral Systems Inc., Albany CA). MCST stimuli were all presented at a visual angle of 4° horizontally and 3.5° vertically, and remained on the screen until the participant responded. The colored geometrical figures were outlined in black against a white background and were identical in luminance.

**Figure 1:**
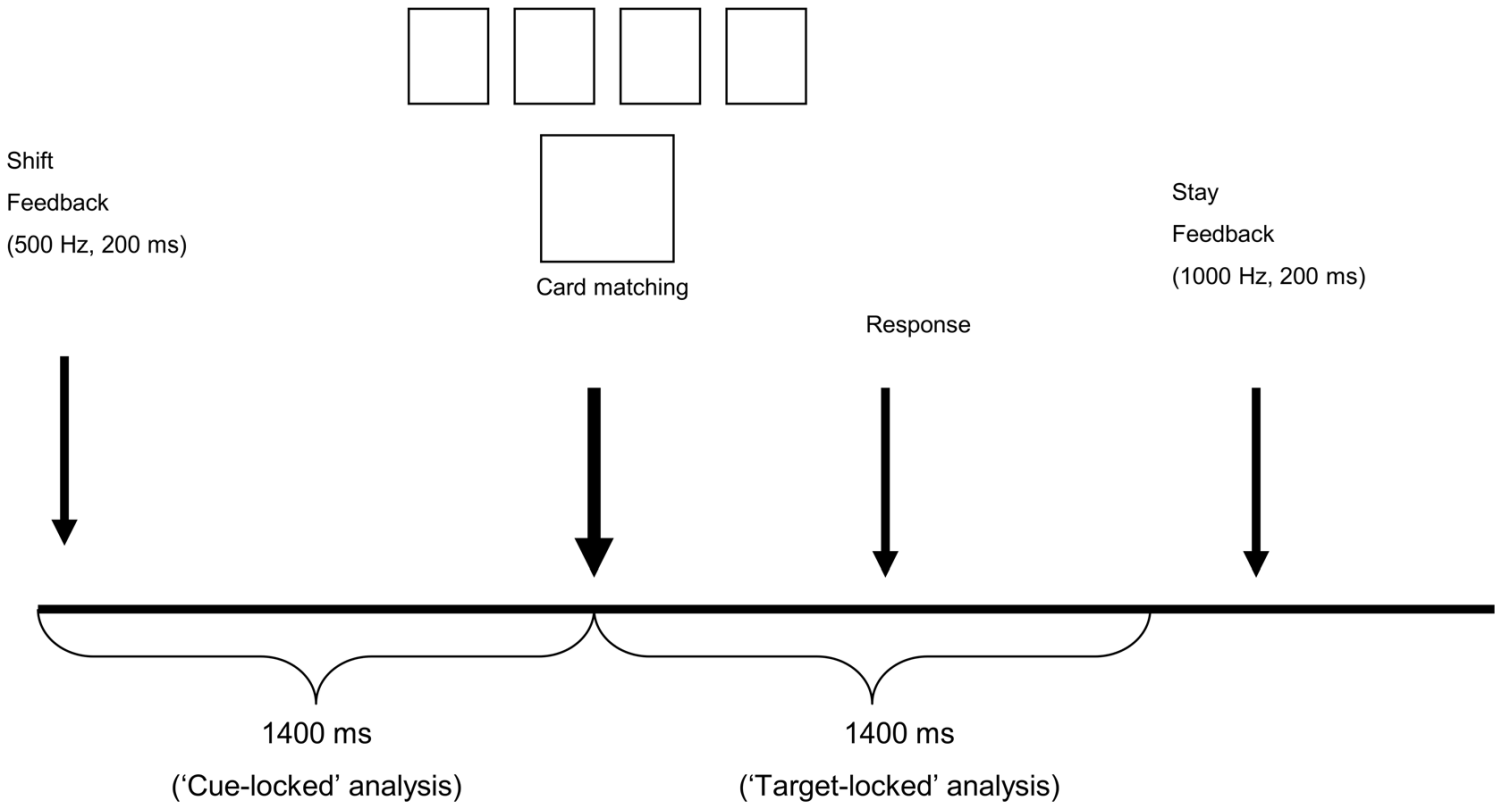
MCST task design and time windows for analysis (modeled after Barcelo, [38]). The data used for the current study's analysis were “cue-locked” only.

MCST cards contain geometrical shapes that differ in three dimensions: color, shape and quantity. Each card can have one of 4 possible characteristics in each dimension (e.g possible colors were red, green, blue and yellow, see figure 1). The MCST stimuli consisted of 24 of the original 62 WCST cards that were to be matched with four target cards (Figure 1). In each trial, subjects had to select which of the 4 target cards matched the single stimulus card below. The trials, totaling 137, were semi-randomly arranged into 18 series, varying in length from 6 to 8 trials per series to avoid rule-switch anticipation.

The instructions to the subjects were to match the stimulus card with one of the target cards (without mentioning how to match them). They had to discover the matching rule (whether according to shape, color, or quantity of elements) by trial and error and then maintain the rule for several repetitions. The only feedback given during the test was whether each matching was right or wrong.

After a varying number of correct responses, the rule changed unpredictably, and the participant had to adjust to a new rule (e.g., from shape to color or number). There was a fixed interval of 1400 msec between the feedback onset and the onset of the next trial. The MCST design employed in this study is depicted in Figure 1 (modeled after [38]).

The MCST included feedback on the subject's performance. A “stay” indicated that the stimulus card was matched with the correct target card (and that the same matching rule was to be used in the next trial). A “shift” cue indicated that a target card was matched incorrectly; indicating that a different matching rule was to be used with the next stimulus card. The cues were delivered 1500–2000 msec after a response (200 msec duration, 10 msec rise/fall times; 65 dB SPL; 1000 Hz for “stay” cues, 500 Hz for “shift” cues).

We defined three types of errors leading to “shift” feedbacks, using Barcelo's terminology [38]:

1. The first erroneous response following a rule change was termed “shift-3D”, as the subject had to handle 3 different dimensions in the working memory (discard the previously used rule and choose between two others).
2. In the trial following the shift-3D cue, the subject had a 50% chance of choosing the correct rule. An incorrect choice would result in a second consecutive shift cue termed “shift-2D”, as the subject now had to handle only two matching rules after rejecting the previous rule.
3. Any additional errors occurring within a series were termed random errors.

Our analysis compared shift-3D and shift-2D errors to random errors in the last 2 trials of a series.

#### 2.2.2. Experimental measures

Mean RTs and mean accuracy were obtained from correct and error trials. Error trials were ones in which the subject failed to select the correct target response when the rule changed, or when the subject failed to follow the instruction to switch/repeat the previous trial [38].

#### 2.2.3 Electrophysiological measures

The EEG was recorded continuously via 64 electrodes mounted on a custom-made cap (Bio-logic Ltd, Claix, France), according to the international 10/20 system [39] and sampled at a rate of 2048 Hz with an analog band pass filter of 0.1 to 70 Hz and 12-bit A/D converter, and stored for off-line analysis. Horizontal eye movements were recorded by electro-oculogram (EOG) electrodes placed on the right and left temples. Vertical eye movements were recorded by an electrode placed under the right eye. All electrode impedances were maintained at or below 5 kΩ.

The EEG was corrected for horizontal and vertical eye movements using Gratton, Coles & Donchin's algorithm (1983) [40] as implemented in the Vision Analyzer (version 1.05) program (Brain Products, Freiburg, Germany), and filtered with a 25 Hz filter. ERP epochs time-locked to “Cues” for early and late errors were averaged separately. An average reference was placed on the chin/nose and was used to reject artifacts. ERPs were epoched into 1400 msec segments with a 200 msec baseline for all conditions. Since we were interested in cognitive processes following an error commission rather than guessing a new rule, only error trials that followed correct responses were segmented and epoched. All epochs were subsequently inspected visually to ensure that they were free of residual artifacts. A grand-average was obtained separately for each condition.

The components were chosen in accordance with the global field power (GFP) measured over the scalp. The time window of each peak corresponded with the time-window observed visually in the GFP for each condition. We used semi-automatic peak detection to mark the ERPs. Since the GFP map demonstrated a fronto-central distribution both components, they were inspected in Fz, FCz and Cz.

#### 2.2.4. Data analyses

Mean RTs and accuracy were obtained only for error trials. Error trials were those in which the participant failed to select the correct response when s/he had to repeat the rule from the previous trial. We included only error trials that followed a correct response. Early errors were considered as an average of errors in the first and second trials whereas late errors were an average of the two last trials (after Barcelo & Knight) [29]. All responses were included in the analyses.

The feedback stage was termed the “cue-locked” condition and card presentation the “target/stimulus-locked” condition (for more details see [38]). In the current study only cue-locked data was analyzed.

**Figure 2:**
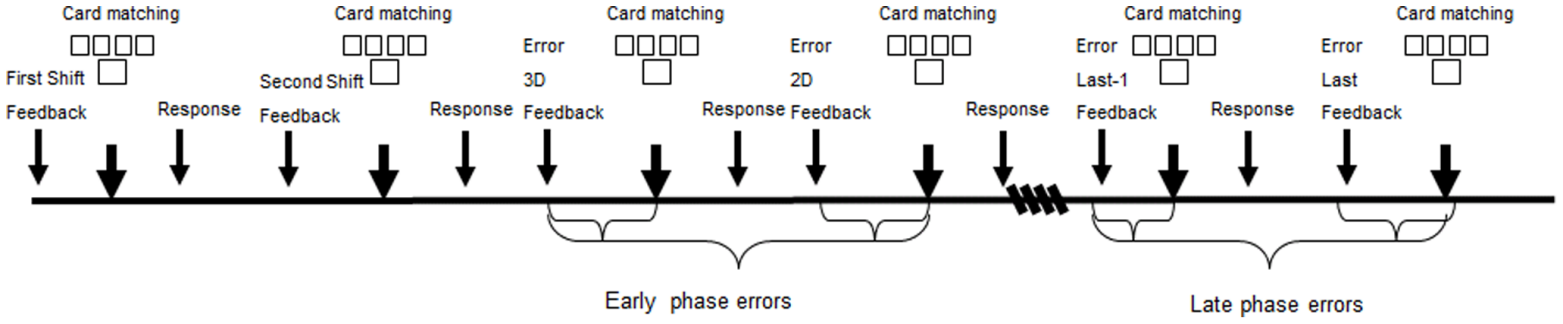
An illustration of the EEG data-analyses.

Prior to data analyses, we verified that there are no significant differences between number of early and late errors using *t*-tests analyses (see Table 2 for details). See Figure 2 for analyses illustration.

**Table 2:** Number of early and late errors among dyslexic and skilled readers adolescents.

	Early errors		Late errors			
	Individuals with dyslexia (A)	Typical readers(B)	Individuals with dyslexia (C)	Typical readers(D)	*P*	Contrasts
Measure	M(SD)	M(SD)	M(SD)	M(SD)		
Number of trials	52(1.57)	48(1.56)	66(2.43)	70(1.69)	1.446, ns	A>B
					0.883, ns	D>C

ns = nonsignificant.

### 2.3 Research design and procedure

All behavioral and electrophysiology testing took place in a dark, quiet room at the participants' school. Participants sat at a distance of about 80 cm from a video display. They were instructed to match the choice-card (target) to one of four presented cards according to one of three rules: the color, shape, or number of objects on the key cards. They were told that when they found the correct rule they would hear a high frequency sound. However, when the rule changed or in a case of an error, they would hear a low-frequency sound. Participants used the index and middle fingers of both hands to press numbers 1 to 4 on the keyboard corresponding to the four presented cards on the screen (the “1” key represented the card on the left of the screen, “2” the second from the left, and so on). The task took about 10 minutes. Participants practiced five series that differed from those employed in the task to make sure they understood the instructions. Speed and accuracy were recorded for all task conditions. The entire task and recordings lasted approximately 45 minutes.

### 2.4. Statistical analyses

Mean error rates and mean reaction times were subjected to two separate two-way repeated-measures analyses of variance (RM-ANOVA) with Phase (early, late errors) as a within-subjects factor and Reading Group (readers with dyslexia, typical readers) as between-subjects factor. Independent *t*-tests were used in order to determine differences error responses between groups.

The mean amplitudes were submitted to two three-way RM-ANOVA with Phase (early, late errors) and Electrodes Site (Fz,FCz,Cz) as the within-subjects variable, and Reading Group (readers with dyslexia, typical readers) as the between-subjects variables for FRN and P300, separately.

In order to find the relations between the cognitive processing following feedback and executive abilities underlying it, Pearson correlation was performed. The relation between cognitive ability underlies cue-evaluation (measured by FRN amplitude) and attentional set shifting (measured by P300) in early and late phases was examined by correlating the differences in FRN and P300 amplitudes for early and late erroneous responses. We also wanted to examine the relations between reading ability and learning and therefore these abilities were also correlated with ERPs using the Pearson correlation.

Bonferroni correction for multiple comparisons was applied for all statistical analyses.

## Results

### 3.1 Background measures

Significant differences were found between individuals with dyslexia and typical readers on reading and cognitive tasks: individuals with dyslexia read fewer words and made more reading errors for words/pseudowords and in oral reading text than typical readers. Individuals with dyslexia exhibited lower digit-span (backward, forward and general), symbol search, coding, and shifting. They also were slower in letters, objects, and RAS naming tasks. The groups were comparable in terms of their reading comprehension ability (see Table 3 for reading ability and Table 4 for cognitive measures).

**Table 3:** A comparison of individuals with dyslexia and typical readers on reading measures (M =  mean, SD =  standard deviation).

		Individuals with dyslexia (A)	Typical readers (B)	*t*	Contrasts
Measures		M (SD)	M (SD)		
One-minute test for words	Speed (number)	57.4 (19.18)	76.6 (21.94)	−3.621**	B>A
	Accuracy (error percentage)	15.78 (6.71)	10.52 (5.38)	3.396**	A>B
One-minute test for nonwords	Speed (number)	26.1 (10.53)	31.32 (12)	0.458 *	B>A
	Accuracy (error percentage)	49.02 (21.33)	25.31 (14.31)	5.162***	A>B
Oral reading	Speed (seconds per word)	0.73 (0.18)	0.56 (0.13)	4.059***	A>B
	Accuracy (error percent)	7.18 (4.54)	3.87 (2.42)	3.462**	A>B
Comprehension (standard score)	Accuracy	0.08 (0.8)	0.55 (0.79)	ns	B>A

*P<.05;

**P<.01;

***P<.001, ns = nonsignificant.

**Table 4:** A comparison of individuals with dyslexia and typical readers on cognitive measures (M =  mean, SD =  standard deviation).

		Individuals with dyslexia (A)	Typical readers (B)	*t*	Contrasts
Measures		M (SD)	M (SD)		
Coding (standard score)	Speed	6.03 (1.93)	8.06 (2.26)	−3.72***	B>A
Symbol search (standard score)	Speed	7.82 (2.76)	10.45 (3.2)	3.405***	B>A
Digit span forward (number of items)	Accuracy	8.07 (1.84)	9.75 (1.58)	−2.507*	B>A
Digit span backward (number of items)	Accuracy	5.39 (1.96)	6.54 (1.62)	−3.844***	B>A
Digit span total (standard score)	Accuracy	6.67 (3.07)	9.21 (2.38)	−3.545**	B>A
Letter naming (seconds)	Speed	29.25 (4.4)	26.48 (3.99)	2.291**	A>B
Object naming (seconds)	Speed	37.53 (6.1)	34.15 (5.86)	2.205*	A>B
RAS naming (seconds)	Speed	30.64 (5.51)	27.39 (5.45)	2.307*	A>B
Trail making — part A (sec)	Speed	30.48 (6.7)	28.96 (9.29)	ns	A>B
Trail making — part A (number of errors)	Accuracy	0.04 (0.2)	0.06 (0.25)	ns	B>A
Trail making — part B (sec)	Speed	87.16(32.58)	65.1(17.4)	3.032**	A>B
Trail making — part B (number of errors)	Accuracy	1.04(2.44)	0.82(1.69)	ns	A>B
Trail making time ratio (A/B)	Speed	2.89(.93)	2.46(1.05)	ns	A>B

*P<.05;

**P<.01;

***P<.001, ns =  nonsignificant.

### 3.2. Behavioral results

#### Accuracy

No differences in errors for the early and late phases between or within the groups were found. See Table 5 for the direction of these results.

**Table 5:** A comparison of individuals with dyslexia and typical readers in terms of accuracy (error rates) and reaction times in the Madrid card-sorting test.

Measure	Early phase		Late phase		*t*	Contrast
	Individuals with dyslexia (A)	Typical readers (B)	Individuals with dyslexia (C)	Typical readers (D)		
Accuracy/% errors – mean (SD)	6.5 (4.55)	5.14 (5.71)	6.65 (5.37)	5.13 (3.71)	ns	
Reaction times in msec – mean (SD)	2327.76 (1213.64)	1985.53 (589.61)	2192.65 (922.4)	1684.72 (365.98)	2.547*	A>B
					2.514*	B>D

*P<.05.

#### Reaction time

Main effect of reading group was observed [*F*(1,53) = 4.618, *P*<.05, η_p_^2^ = .08], indicating longer RTs for individuals with dyslexia than for typical readers. A main effect of phase was observed [*F*(1,53) = 5.791, *P*<.05, η_p_^2^ = .099], with longer RTs for early vs. late errors. No significant phase X reading group interaction was found. Data are presented in Table 5.

### 3.3. Electrophysiology

#### 3.3.1. FRN component

**Figure 3:**
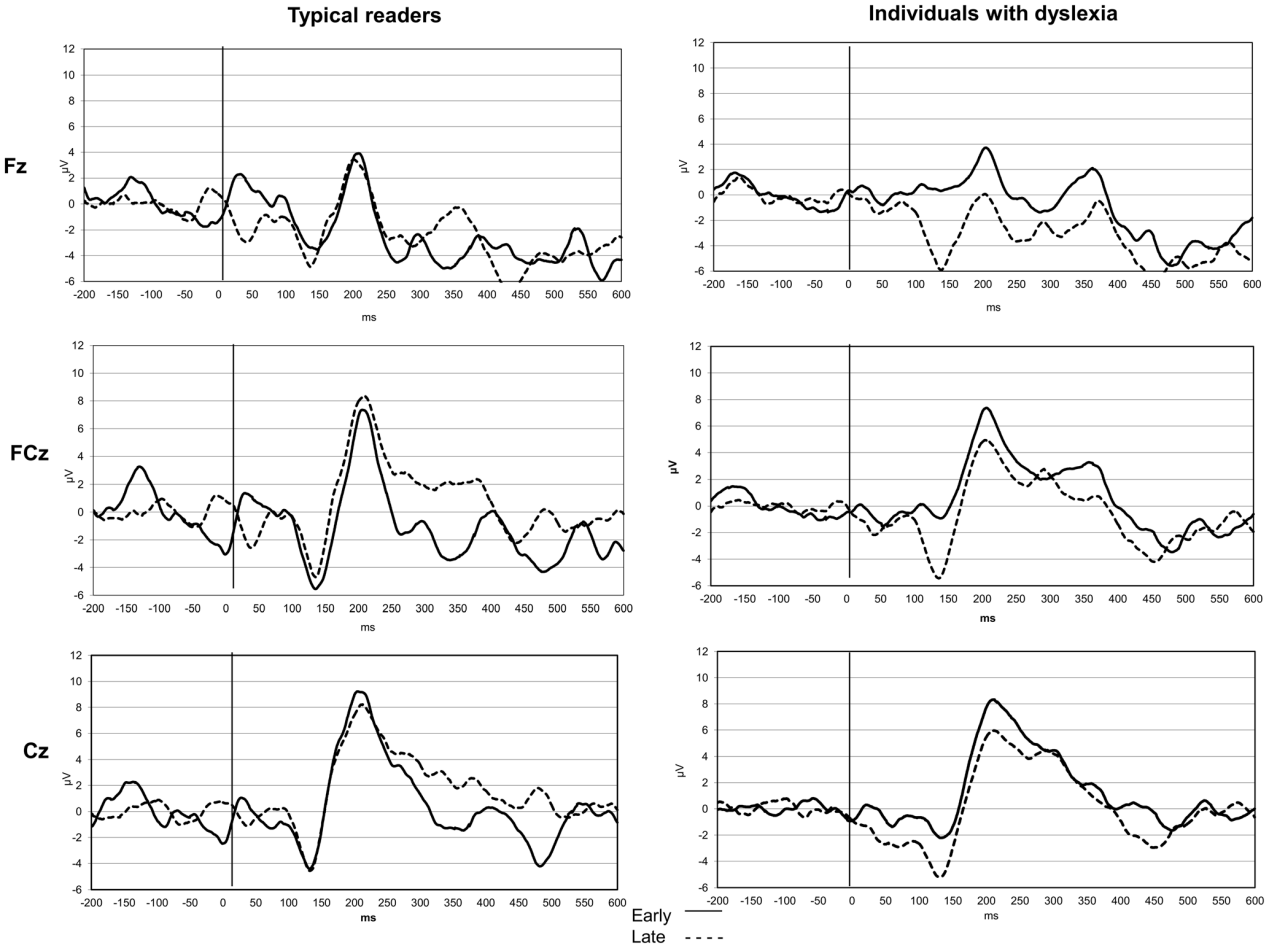
FRN and P300 cue-locked Event-related components. Elicitation of ERPs following cue presentation for individuals with dyslexia (right column) and typical readers (left column). Upper rows: frontal region (Fz electrode); middle rows: fronto-central regions (FCz electrode) bottom rows: central regions (Cz electrode). Smooth line: “early phase”; dashed line: “late phase.” FRN was identified as the most negative peak at 100–250 msec and P300 as the most positive peak at 250–400 msec. X axis: time in msec; Y axis: amplitude in µV. Note that the negative axis is plotted down.

FRN components were defined as the most negative deflections occurring between 100 and 250 msec after the feedback. In order to explore whether differences in early and late phase conditions are reflected also in FRN components, several RM-ANOVAs were performed. A main effect of reading group was found [*F*(1,56) = 4.799, *P*<.05, 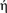
_p_^2^ = .079] at the Cz electrode, indicating a slightly smaller FRN in individuals with dyslexia as compared to typical readers. A main effect of phase was also observed [*F*(3,54) = 3.722, *P*<.05, 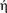
_p_^2^ = .171] in all electrodes; this resulted from a larger FRN for the late phase versus the early phase. The significant phase × reading group interaction [*F*(3,54) = 3.517, *P*<.05, 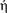
_p_^2^ = .163] pointed at slightly larger differences between FRN for early versus late phases in individuals with dyslexia than in typical readers. Within-subjects RM-ANOVA analysis suggested that this main effect resulted from larger FRN for late than for early phases only in individuals with dyslexia [*F*(3,24) = 9.291, *P*<.001, 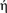
_p_^2^  = .537]. See Table 6 and Figure 3 for details.

**Table 6:** Amplitudes (in µV) of FRN and P300 cue-locked components for individuals with dyslexia and typical readers for early and late errors conditions.

		Early errors		Late errors			
		Individuals with dyslexia (A)	Typical readers (B)	Individuals with dyslexia (C)	Typical readers(D)	*t*	Contrast
		M(SD)	M(SD)	M(SD)	M(SD)		
Component	Electrode						
FRN	Fz	−2.73(5.22)	−6.7(5.29)	−7.47(6.69)	−6.64(5.15)	2.859**	B>A
						ns	C>D
						−3.458**	C>D
	FCz	−4.77(6.11)	−7.91(6.78)	−7.6(4.5)	−7.87(7.06)	1.838*	B>A
						ns	D>C
						2.204*	C>A
	CZz	−2.99(4.1)	−6.08(3.72)	−6.39(3)	−6.25(4.26)	2.996**	B>A
						ns	C>D
						−3.12**	C>A
P300	Fz	4.2(4.02)	7.66(6.53)	2.37(4.8)	7.16(5.49)	−2.385*	B>A
						−3.513**	D>C
	FCz	9.5(6.77)	10.42(7.78)	6.89(5.7)	11.58(9.43)	−2.245*	D>C
						ns	B>A
						−2.203*	A>C
	Cz	10.53(5.04)	11.45(5.87)	8.35(6.42)	11.81(6.99)	−1.948*	D>C
						ns	F>E

*P<.05;

**P<.01, ns =  nonsignificant.

#### 3.3.2. P300 component

P300 was defined as the most positive deflection occurring between 250 and 400 msec after a feedback. We examined whether the same effect of phase (early vs late) was observed in P300 component. Main effect for group was observed [*F*(3,54) = 5.372, *P*<.05, 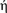
_p_^2^ = .23], implying smaller P300 amplitudes in individuals with dyslexia compared to typical readers (Table 6 and Figure 3). No significant main effect for within-group analyses was found.

To summarize, both groups exhibited larger FRN amplitudes for late compared to early phases. However, the difference between early and late phases was more pronounced in individuals with dyslexia. This was accompanied by a trend of shorter RTs and decreased error rates in the late phase. P300 amplitudes decreased more in individuals with dyslexia than in typical readers. The relations between the FRN and P300 were demonstrated by the positive correlation in the late phase (for Cz electrode: r = .394, *P*<.01, for Fz electrode: r = .368, *P*<.01) indicating that larger FRNs were associated with smaller P300s. Changes between early and late FRN amplitudes were correlated with changes in P300 in all participants (r = .461, *p*<.001).

### 3.4. Correlations

Correlations between P300 and reading measures are given in table 7. The correlation of P300 for the late phase with reading measures showed that greater P300 amplitudes were associated with more accurate and fluent reading. Faster speeds of processing and better memory measures were associated with higher accuracy scores and faster words/pseudowords and oral reading.

**Table 7:** Correlations of ERPs and cognitive abilities with reading measures.

Cognitive ability	Measure	Reading measures	Correlation (r)
Attention set shifting	P300 amplitude in the late phase (µV)	Number of correct words read per minute	.34**
		Oral reading time (sec)	.27*
		Reading comprehension score	.26*
Speed of processing	Object naming time (sec)	Number of erroneous words per minute	.33**
		Oral reading time (sec)	.45***
	Symbol search standard (standard score)	Number of correct words read per minute	.466***
		Number of correct pseudowords read per minute	.27*
		Oral reading time (sec)	-.43***
			
Memory	Digit span (standard score)	Number of erroneous words per minute	-.41**
		Number of correct pseudowords per minute	.3*
		Oral reading time (sec)	-.38*

*P<.05;

**P<.01;

***P<.001. Data were corrected for multiple comparisons.

## Discussion

The current study aimed to examine learning within a series among adolescents with dyslexia during the MCST.To do so, we examined performance on the MCST using FRN and P300. Learning was assessed as a function of change in ERP components and in behavioral performance between early and late phases of individual series within the task. To the best of our knowledge, this is the first study to evaluate these components within MCST series in individuals with dyslexia.

### Behavioral measures

In line with our hypothesis, the main behavioral difference between both groups was in reaction times (RTs). Individuals with dyslexia exhibited longer RTs than typical readers. In the MCST, a change in rule requires the participant to shift attention to a different characteristic of the stimulus, to keep it in the working memory and to inhibit the previous response [27]. Speed of processing plays an important role in such activities: when speed of processing is slow, the working memory is more likely to be overloaded and the attention required is higher [41]. We therefore suggest that slower RTs achieved by readers with dyslexia represent reduced speed of processing. This has already been demonstrated in this group of readers [5].

The fact that both groups showed similar accuracy rates is of potential interest, as one would expect typical readers to perform better than their peers with dyslexia. While performing the task, individuals with dyslexia may have recruited brain regions outside the prefrontal cortex to compensate for their executive deficit [19] such as parietal regions involved in cognitive control (see the dual-network model [42]). Furthermore, the MCST relies on spatial abilities (i.e., the participant has to recognize the characteristics of a stimulus card and to categorize it), which localize to the right hemisphere [43]. Functional magnetic resonance imaging would be a suitable tool to characterize the entire network contributing to MCST performance in individuals with dyslexia.

Participants in both groups showed decreased error rates in late phases of individual series, which confirmed our hypothesis. Yet, the differences between early and late phases did not reach statistical significance in either group. Previous studies (e.g [29]) have shown a significant reduction in errors during late phases, when adults performed the test. The relative absence of effect can be attributed to the fact that executive functions assessed by the MCST localize to frontal brain regions, which complete maturation in the late 20s [44]. In the current study, participants in both groups were young adolescents, who might have not been able to perform at an adult level. This is a possible limitation of the current study.

### Electrophysiological measures

Our second hypothesis was that in early phases of individual series, individuals with dyslexia would exhibit decreased FRNs and P300 amplitudes compared to typical readers. The results of the current study confirm our hypothesis: individuals with dyslexia showed decreased FRNs in the early phase, which then increased to levels comparable to those of typical readers towards the end of a series. We suggest this increase might represent a distinct learning pattern that occurs preferentially in individuals with dyslexia with repetition of a task.

This pattern of “normalization” of an ERP component related to the error monitoring system has been observed in previous studies dealing with individuals with dyslexia [6], [7], [45], [46], [47]. These studies utilized the Error-Related Negativity (ERN) as a marker for the function of this system. Following a working memory intervention, adults with dyslexia demonstrated a similar increase of ERN amplitudes to typical reader levels that was associated with improved working memory measures [7]. The possible association between these findings is intriguing in light of the commonalities between the ERN and the FRN: both are part of the learning circuit [25], [48] and increase in amplitude preferentially in response to feedback stimuli associated with unfavorable outcomes (in our case — an erroneous choice of a card) [49]. According to this hypothesis, FRN is the cue-locked variant of the response-locked ERN. The fact that the interaction between these components seems to depend on the specific task further complicates any clear conclusions at this time. Examination of ERN and FRN characteristics in the same group of individuals with dyslexia could fill this knowledge gap.

The control group of typical readers showed only minor reductions in FRN amplitudes. Previous studies have shown a general reduction in FRN throughout the performance of a task [23], [24], [25]. These apparent inconsistencies might be related to the nature of the current study task. We have assessed learning within a series of the MCST rather than throughout the entire test. It is possible that the learning effect during a repetition of a single rule was not large enough to produce an electrographic change. However, the current study focused on short-term rather than long-term learning-related changes in performance, which were expected to be more limited than the changes observed in studies examining learning in its more conventional sense. Yet, it would be interesting to examine whether a card-sorting task consisting of longer series will have a larger impact on these measures.

An analogous phenomenon was observed in P300 amplitudes during individual MCST series. During early phases, individuals with dyslexia showed smaller P300 amplitudes compared to typical readers. However, during late phases individuals with dyslexia showed a further decrease in P300 amplitudes, while amplitudes among typical readers remained essentially constant throughout a series. Larger P300 amplitudes were associated with increased reading and cognitive abilities.

In the process of learning a task, P300 amplitudes were expected to decrease [23], [24], as the amount of novelty diminishes and the attentional resources required by the task decrease [50]. As was postulated in our third hypothesis, such a decrease showed a significant correlation with the increase in FRN amplitudes in the study group, which strengthen the correspondence between FRN and P300 as a “complex”. These results imply that repetition of a rule within a series had a beneficial effect on individuals with dyslexia, but not on typical readers. In contrast to FRN, the amplitude difference between early and late phases rather than the absolute values are of significance in the case of individuals with dyslexia.

We suggest that the current results may represent a rapid compensation phenomenon that leads to more efficient performance during late phases of MCST series. Even repetition of a single matching rule resulted in decreased RTs as well as in ERP changes related to learning mechanisms. While these changes were insufficient to lead to decreased error frequency, the stable accuracy rates rule out increased impulsivity as a possible explanation for the reduced RTs.

These results do not imply that individuals with dyslexia have superior capabilities to adjust performance based on errors compared to typical readers. As exemplified by the changes in FRN amplitudes, they merely show that the lower one's baseline, the higher the chance to benefit from an appropriately designed intervention. Based on our results, we hypothesize that in the case of individuals with dyslexia, the lower the baseline performance in a given task, the larger the potential for rapid improvement following a targeted intervention.

In summary, the current study supports previous findings associating dyslexia with a top-down processing impairment [16].It also highlights the role of neuroimaging in providing information about cognitive mechanisms. From a clinical point of view, our findings support the notion that repetitions and rehearsals of a task seem to benefit these adolescents almost immediately. This may encourage educators and parents to repeat instructions and learning materials for students with dyslexia.
